# Supplementary material for: Heavy Ion-Responsive lncRNA EBLN3P Functions in the Radiosensitization of Non-Small Cell Lung Cancer Cells Mediated by TNPO1
Source: Cancers (Basel). 2023 Jan 13;15(2):511. doi: 10.3390/cancers15020511 (PMC9856274; doi:10.3390/cancers15020511)
Supplement: Supplementary file 1 [file cancers-15-00511-s001.zip › cancers-2099457-supplementary.pdf]

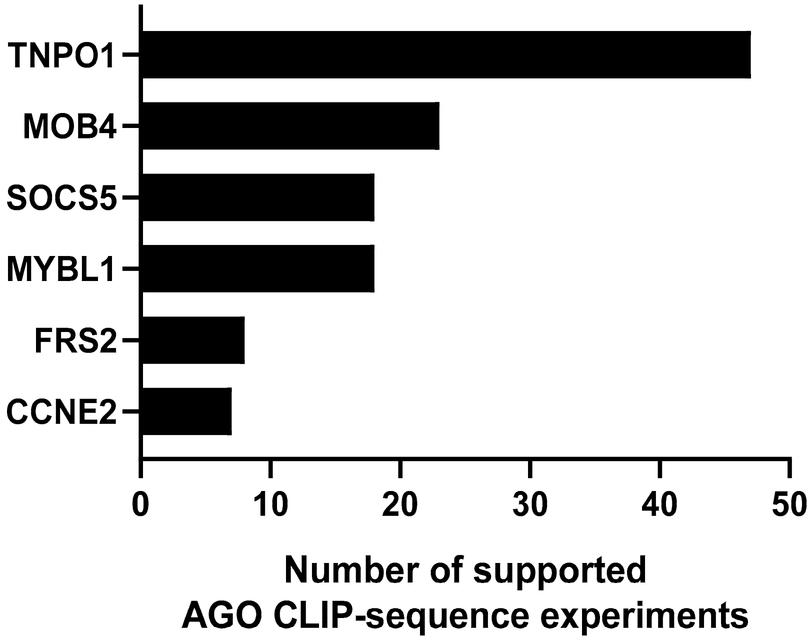

Figure S1. Number of the reported AGO-CLIP experiments was analyzed to predict the interaction between miR-144-3p and its target genes. Data are from ENCORI database (https://starbase.sysu.edu.cn/).

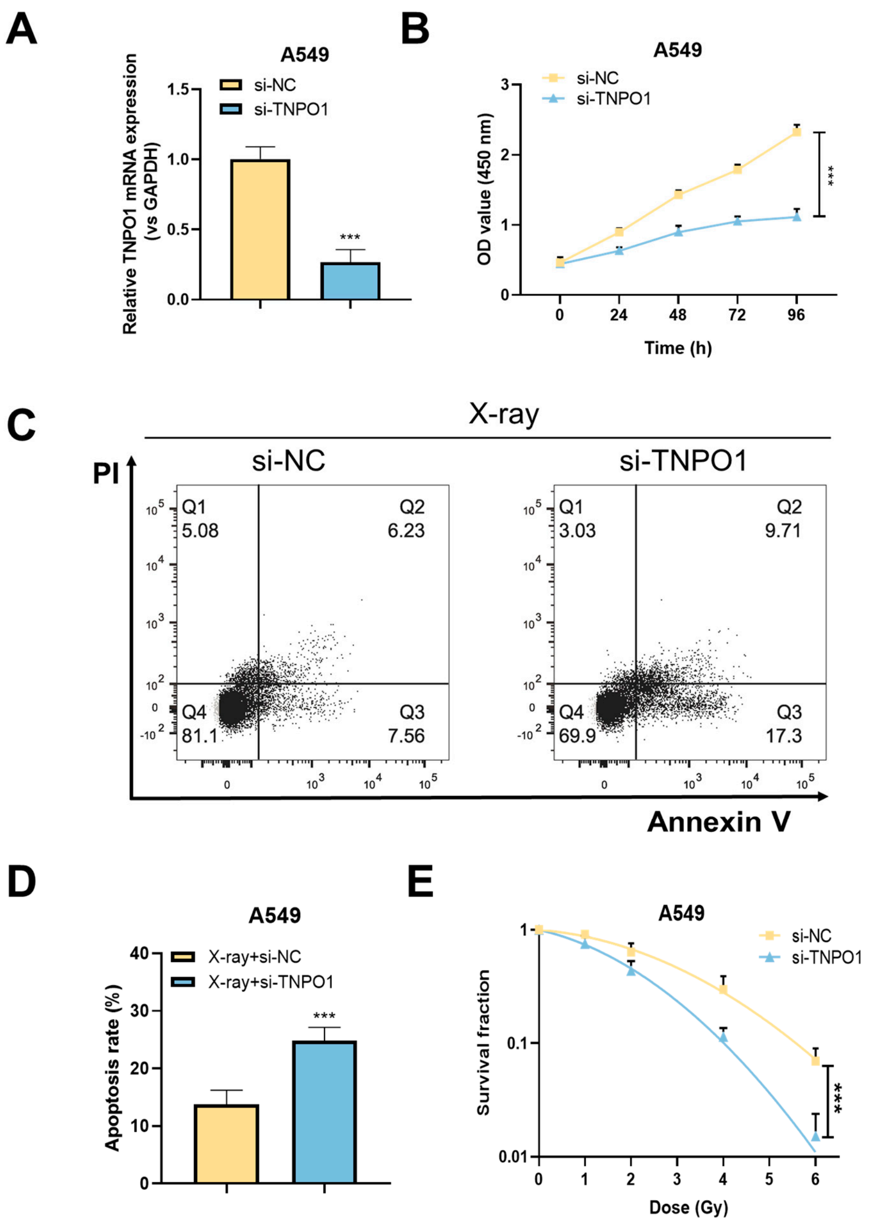

Figure S2. TNPO1-knockdown inhibited proliferation of A549 cells and sensitized the cells to X-ray irradiation. (A) The relative expression of TNPO1 in the cells transfected by si-TNPO1 was verified by qRT-PCR. (B) The cell viability of A549 cells with TNPO1-knockdown was detected by CCK-8 assay. (C-D) The apoptosis rate of A549 cells with TNPO1-knockdown after exposure to X-ray irradiation. (E) The survival fraction of A549 cells with TNPO1-knockdown was determined by colony formation assay. ***$p < 0.001$.

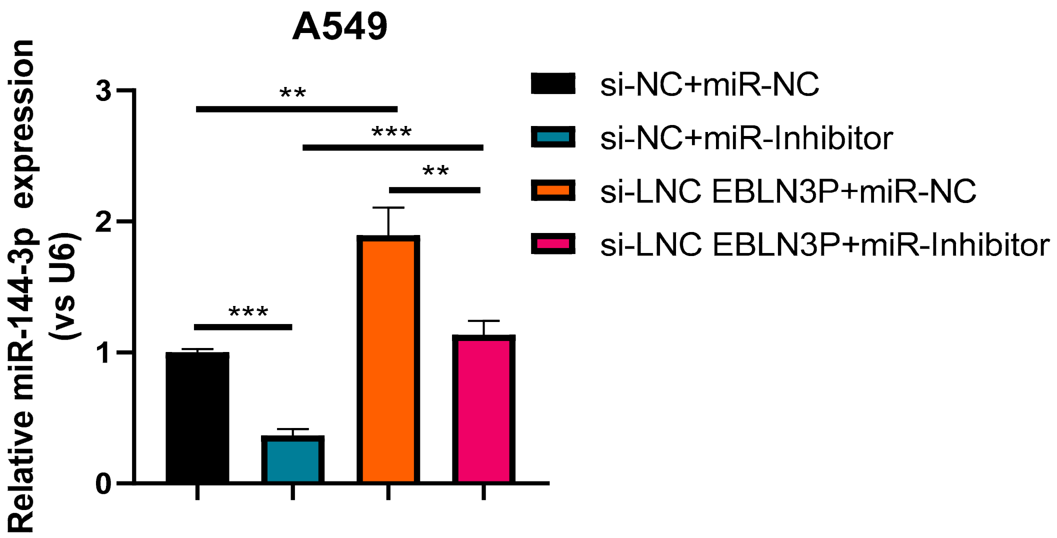

Figure S3. The relative expression of miR-144-3p in A549 cells co-transfected with si-LNC EBLN3P and miR-Inhibitors was determined by qRT-PCR. **$p < 0.01$, ***$p < 0.001$.

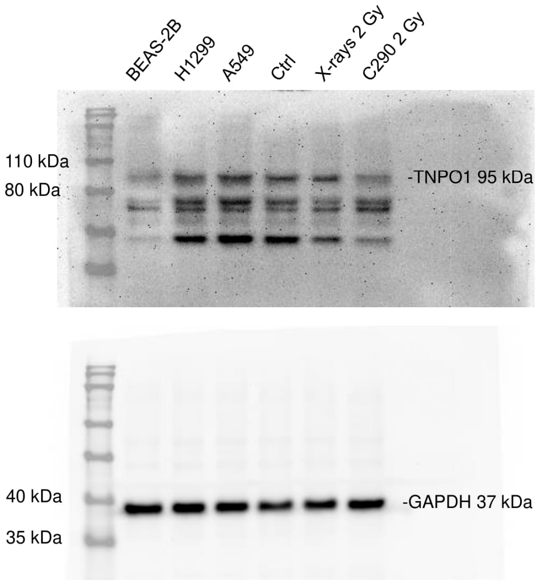

Figure S4: The whole blot image of the western blotting data of Figure 2.

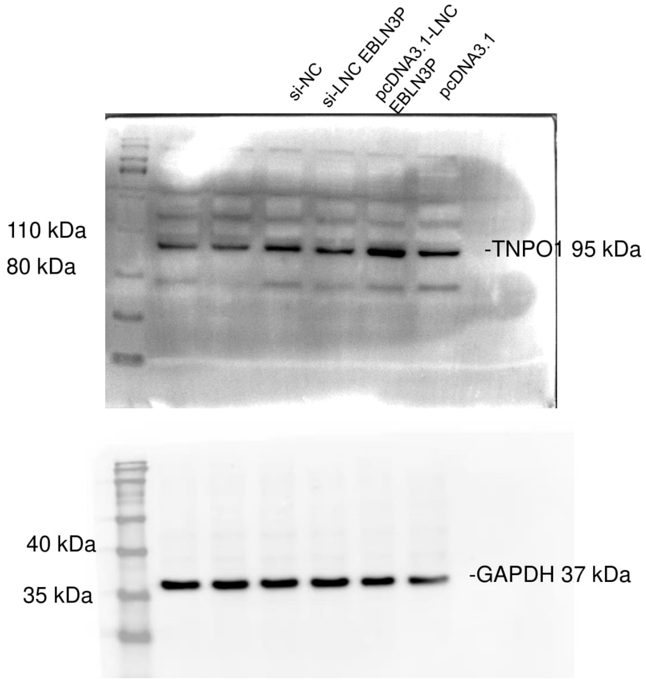

Figure S5: The whole blot image of the western blotting data of Figure 4.
